# ABO blood types and sepsis mortality

**DOI:** 10.1186/s13613-021-00844-2

**Published:** 2021-04-20

**Authors:** Theis S. Itenov, Daniel I. Sessler, Ashish K. Khanna, Sisse R. Ostrowski, Pär I. Johansson, Christian Erikstrup, Ole B. Pedersen, Sofie L. Rygård, Lars B. Holst, Morten H. Bestle, Lars Hein, Anne Lindhardt, Hami Tousi, Mads H. Andersen, Thomas Mohr, Jens D. Lundgren, Jens-Ulrik Jensen

**Affiliations:** 1 CHIP / PERSIMUNE, Department of Infectious Diseases, Rigshospitalet, Copenhagen University Hospital, Blegdamsvej 9, 2100 Copenhagen, The Capital Region of Denmark Denmark; 2 Department of Outcomes Research, Anesthesiology Institute, Cleveland Clinic, Cleveland, OH USA; 3 Wake Forest University School of Medicine, Wake Forest Baptist Health, Winston-Salem, NC USA; 4 Department of Clinical Immunology, Rigshospitalet, Copenhagen University Hospital, Copenhagen, Denmark; 5 Department of Clinical Immunology, Aarhus University Hospital, Aarhus, Denmark; 6 Department of Clinical Immunology, Næstved Sygehus, Næstved, Denmark; 7 Department of Intensive Care, Rigshospitalet, Denmark; 8 Department of Anaesthesiology, Nordsjællands Hospital, Hillerød, Denmark; 9 Department of Anaesthesia and Intensive Care, Bispebjerg and Frederiksberg Hospital, University of Copenhagen, Copenhagen, Denmark; 10 Department of Anaesthesiology, Herlev Hospital, Copenhagen, Denmark; 11 Department of Anaesthesiology, Aarhus University Hospital, Aarhus, Denmark; 12 Department of Anaesthesiology, Gentofte Hospital, Hellerup, Denmark; 13 Respiratory Section, Department of Internal Medicine, Gentofte Hospital, Copenhagen University Hospital, Copenhagen, Denmark; 14 Outcomes Research Consortium, Cleveland, OH USA; 15 Department of Clinical Medicine, University of Copenhagen, Copenhagen, Denmark

**Keywords:** Anaesthesia, Blood type, Mortality, Sepsis, Septic shock, Intensive care

## Abstract

**Background:**

We aimed to determine if the ABO blood types carry different risks of 30-day mortality, acute kidney injury (AKI), and endothelial damage in critically ill patients with sepsis. This was a retrospective cohort study of three independent cohorts of critically ill patients from the United States and Scandinavia consisting of adults with septic shock. We compared the 30-day mortality across the blood types within each cohort and pooled the results in a meta-analysis. We also estimated the incidence of AKI and degree of endothelial damage, as measured by blood concentrations of soluble thrombomodulin and syndecan-1.

**Results:**

We included 12,342 patients with severe sepsis. In a pooled analysis blood type B carried a slightly lower risk of 30-day all-cause mortality compared to non-blood type B (adjusted HR 0.88; 95%-CI 0.79–0.98; *p* = 0.02). There was no difference in the risk of AKI. Soluble thrombomodulin and syndecan-1 concentrations were lower in patients with blood type B and O compared to blood type A, suggesting less endothelial damage.

**Conclusion:**

Septic patients with blood type B had less endothelial damage, and a small reduction in mortality. The exposure is, however, unmodifiable.

**Supplementary Information:**

The online version contains supplementary material available at 10.1186/s13613-021-00844-2.

## Introduction

ABO blood types arise from variations in an oligosaccharide molecule on the H-antigen which is present on both the surface of erythrocytes and endothelial cells. Variations in oligosaccharide composition are controlled by the gene coding a glycosyl-transferring enzyme [1, 2]. Endothelial damage, including disruption of the glycocalyx, increases the risk of organ failure and death in critically ill patients, and it has been proposed to play a pivotal role in the pathophysiology of critical illness associated organ dysfunction [3–5].

Sepsis is frequently complicated by organ dysfunction, including acute kidney injury (AKI) and acute respiratory distress syndrome (ARDS) [6, 7], both of which increase the risk of dying. ABO blood type A seems to increase the risk of sustaining AKI and ARDS in patients with either sepsis or trauma [8, 9]. However, it remains unclear whether ABO blood type influences sepsis-related mortality, much less what the mechanism might be [8–11].

Our overall goal was to determine if ABO blood type influences 30-day mortality, AKI, and endothelial damage in septic adults. In an initial cohort of sepsis patients from the Procalcitonin And Survival Study trial (described below), we tested the hypothesis that sepsis survival is worse in patients with blood type A. Based on these initial results, we refined the hypothesis. Specifically, we thereafter tested the primary hypothesis that 30-day mortality from sepsis and/or septic shock is lower in patients who have blood type B than other blood types. Secondarily, we tested the hypothesis that soluble thrombomodulin and syndecan-1 concentrations are lower in patients with blood type B than other blood types, suggesting less endothelial damage. This refined hypothesis was tested in two new cohorts of patients with sepsis and/or septic shock, and we here present results from all cohorts.

## Materials and methods

We included patients from three independent cohorts: (1) The Procalcitonin And Survival Study (PASS) ICU cohort 2006–2009; (2) Transfusion Requirements in Septic Shock (TRISS) trial 2011–2013, and (3) patients from the Cleveland Clinics Main Campus medical ICU (CCF MICU) 2009–2019. We included adults with sepsis diagnosed per current consensus definitions of sepsis or septic shock, except in the TRISS cohort where the 2001 definition was used [12, 13].

In brief the Procalcitonin And Survival Study (PASS) study ICU cohort enrolled adults admitted to Danish ICUs between 2006 and 2009. Details on inclusion and intervention in the PASS study have been previously presented [14]. From the PASS cohort we included all patients with a blood type available via any Danish blood bank and sepsis according to the sepsis-3 definition [12]. All patients or legal representatives gave written informed consent. The study was approved by the local ethical committee (ref. no.: H-KF-272-753).

The Transfusion Requirements in Septic Shock (TRISS) trial enrolled adults with septic shock and a haemoglobin level of ≤ 9 g/dL admitted to Scandinavian ICUs between 2011 and 2013. The TRISS trial inclusion criteria and interventions have been previously presented [15]. We included all patients from the TRISS cohort who had a blood type available. The TRISS study was approved by the local ethical committee (ref. No.: H-3-2011-114).

Finally, we included adults admitted to the medical ICU of the Cleveland Clinic Foundation Main Campus (CCF MICU) between 2009 and 2019, with sepsis defined according to the sepsis-3 definition. Extraction of data was approved by the institutional review board, and the need for individual consent was waived.

ABO blood types were ascertained from local blood banks or laboratories using standard clinical methods.

Death was quantified as all-cause mortality within 30 days of study inclusion in the individual studies. AKI was defined according to the creatinine criteria of KDIGO classification [16]. Specifically, increase in SCr by ≥ 0.3 mg/dl (≥ 26.0.5 mmol/l) within 48 h; or increase in SCr to ≥ 1.5 times baseline, as previously applied [17]. In patients without a pre-admission creatinine measurements, concentrations were estimated using the 4-variable Modified Diet in Renal Disease (MDRD) [18].

Endothelial damage was estimated by the serum concentration of soluble thrombomodulin (sTM) and syndecan-1 in the PASS cohort. Serum was sampled on study inclusion in standard serum separation tubes. After separation the isolated plasma and serum were frozen at − 80 °C within 5 h for subsequent analysis. The samples underwent one freeze/thaw cycle before analysis for aliquoting. Both were measured in serum using an enzyme-linked immunosorbent assay (Diaclone SAS, Besancon, France).

## Statistics

The statistical analysis was conducted according to a pre-specified statistical analysis plan. The distribution of blood types among the cohorts was compared with a Chi-square test.

Time to event outcomes was analysed by Cox regression in the individual cohorts. The proportional hazards assumption was confirmed by inspection of scaled Schoenfeld residuals, and test for independence between residuals and time. ABO blood types are coded genetically and unaltered by clinical interventions. Further, no current medical or surgical treatments except transfusion and donor–recipient transplant matching are guided by the ABO blood types. Baseline differences in disease severity or comorbidity might therefore be either incidental or directly consequent to ABO blood type, but the risk of confounding seems low.

We adjusted all models for age, sex, known ischemic heart disease, previous stroke and whether patients had a pulmonary infection or not. We tested the models for interaction effects between pulmonary infection and need for mechanical ventilation. The interaction was only included in the final model if significant. Age was allowed non-linear effects using restricted cubic splines with 3 knots if this significantly improved model fit compared to a linear effect [19]. The model fit was assessed by inspection of martingale residuals. Finally, the model was checked for overly influential observations by inspection of deviance residuals.

Pooling of results among the three studies was done by merging the three cohorts and applying a Cox model stratified for centre and adjusted for age, sex, known ischemic heart disease, previous stroke and whether patients had a pulmonary infection or not. This allows the baseline hazard to vary between the cohorts and thus account for between-study heterogeneity in baseline hazard [20]. To assess the between-study variation the I^2^ statistics are presented, and we tested for significant heterogeneity using the Cochran Q-test.

The risk of AKI was analysed by standard multiple logistic regression. The same considerations regarding confounding and effect of age as in the previous analyses apply.

The degree of endothelial damage was analysed with standard least squares linear regression. The same considerations regarding confounding as the aforementioned analyses apply, and the models were adjusted for age, sex, known ischemic heart disease, previous stroke and whether patients had a pulmonary infection or not. The models were assessed for non-linearity, normality of residuals and heteroskedasticity using standard statistical methods.

Missing data were rare, and patients with missing covariates were excluded from the analysis. Continuous markers are presented as median (IQR) and categorical markers as number (%) and compared with Mann–Whitney U test and Chi-square test as appropriate. Statistical significance was set at *P* < 0.05. All analyses were completed with R, version 3.3.3 [21].

## Results

**Fig. 1:**
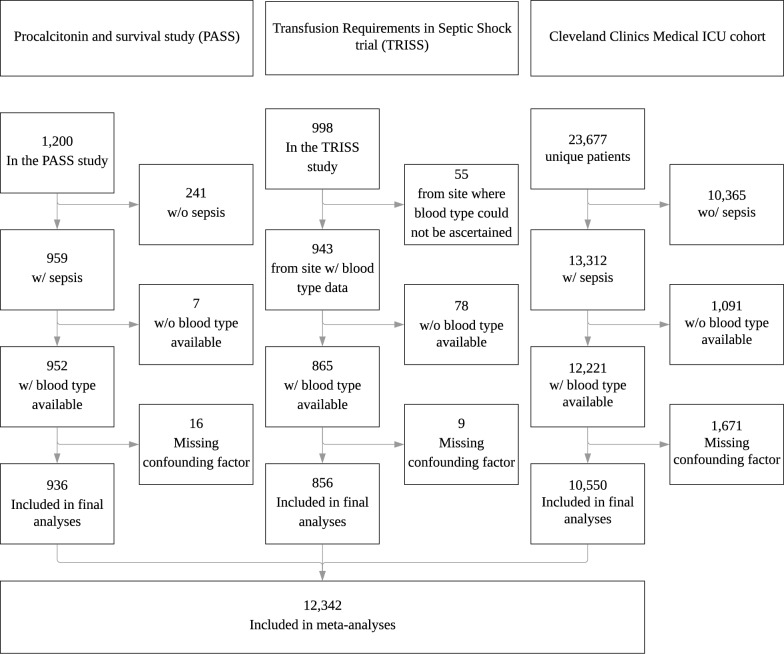
Patient inclusion

A total of 12,342 patients were included in the analysis: 936 from PASS, 856 from TRISS, and 10,550 from the Cleveland Clinic (Fig. 1). Table 1 presents baseline characteristics in the three cohorts. The distribution of ABO-types corresponded to the distribution expected in a Caucasian population [22]. There were no difference in the proportions of blood types between the PASS and TRISS cohorts (*P* = 0.74), while the Cleveland Clinic had fewer patients with blood type A and more with O and B than the PASS (*P* < 0.001) and TRISS cohort (*P* = 0.003) (Table 1).

**Table 1:** Baseline characteristics in the study cohorts

Characteristic	Procalcitonin and survival study (PASS) (*n* = 936)		Transfusion requirements in septic shock (TRISS) trial (*n* = 856)		Cleveland clinics medical ICU cohort (*n* = 10,550)	
Median age—years (IQR)	67 (58–76)		66 (58–74)		64 (54–74)	
Male sex—no. (%)	515 (55)		390 (46)		5502 (52)	
Median BMI—index (IQR)	25 (22–28)				28 (23–34)	
Median weight—kg (IQR)	75 (63–87)		75 (64–87)		80 (66–98)	
Blood type—no. (%)						
A	420 (45)		368 (43)		3942 (37)	
B	102 (11)		95 (11)		1523 (14)	
AB	39 (4)		31 (4)		433 ( 4)	
O	375 (40)		362 (42)		4652 (44)	
Diabetes—no. (%)	138 (15)		–		4552 (43)	
Hypertension—no. (%)	168 (18)		–		7109 (67)	
Charlson Score ≥ 2—no. (%)	333 (36)		–		8828 (84)	
Median SOFA—score (IQR)	7 (4–9)		10 (8–12)		6 (4–9)	
Mechanical ventilation—no. (%)	629 (67)		611 (71)		1356 (13)	
Median creatinine µmol/L (IQR)	127 (80–215)		131 (79–203)		129 (80–241)	
Renal replacement therapy—no. (%)	152 (16)		103 (12)		148 ( 1)	
Median urinary output—mL/kg/h (IQR)	1.1 (0.5–1.7)		–			
Median MAP—mmHg (IQR)	71 (61–84)		58 (52–63)		63 (54–72)	
Vasopressor treatment—no. (%)	433 (46)		765 (89)		2330 (22)	
Inotropic treatment—no. (%)	201 (22)		–		–	
Septic shock—no. (%)	603 (64)		856 (100)		4714 (55)	
Median platelet count— × 10^6^/L (IQR)	234 (148–333)		–		165 (93–244)	
Median WBC—× 10^6^/L (IQR)	13 (9–19)		–		–	
Median bilirubin—µmol/L (IQR)	10 (5–18)		–		12 (7–27)	

### ABO blood type and 30-day mortality

**Fig. 2:**
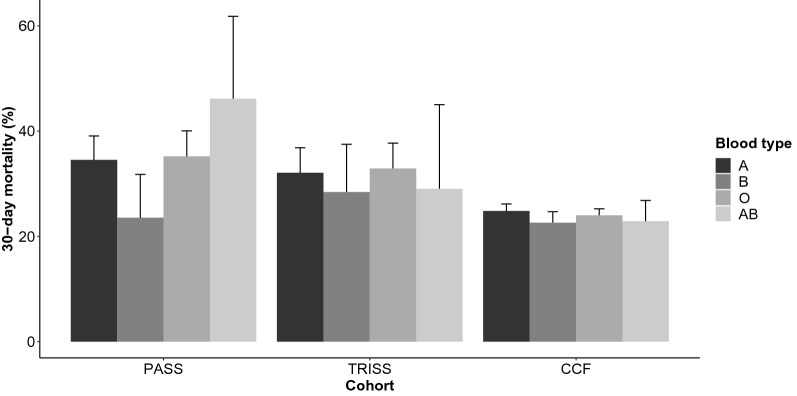
Crude 30-day mortality in three independent ICU cohorts

Thirty-day all-cause mortality in the cohorts were PASS 319/936 (34%), TRISS 273/856 (32%) and CCF 2537/10,550 (24%). The B blood type had numerically lower risk of death in the PASS cohort (Fig. 2). Based on this observation, we compared blood type B vs. the other blood types in all further analyses. We consistently found that the B blood type had a lower mortality compared to the other blood types in both unadjusted and adjusted analyses, although only reaching statistical significance in the PASS cohort (Table 2). We did not detect an interaction between the need for mechanical ventilation and the effect of the blood type, nor whether the patients had a pulmonary infection or not.

**Table 2:** Effect of blood type on mortality in the three ICU cohorts—Cox regression

Blood type (percentage of cohort)	Univariable			Multivariable^a^		
	HR	(95%-CI)	P	HR	(95%-CI)	P
*PASS cohort*						
A (45%)	1.00			1.00		
B (11%)	0.64	(0.42–0.99)	0.05	0.63	(0.41–0.97)	0.03
AB (4%)	1.61	(0.98–2.62)	0.06	1.59	(0.97–2.61)	0.06
O (40%)	1.04	(0.82–1.32)	0.74	1.00	(0.79–1.27)	0.99
Non-B (89%)	1.00			1.00		
B (11%)	0.61	(0.41–0.94)	0.02	0.61	(0.40–0.93)	0.02
*TRISS cohort*						
A (43%)	1.00			1.00		
B (11%)	0.87	(0.57–1.32)	0.51	0.91	(0.60–1.38)	0.66
AB (4%)	0.92	(0.47–1.81)	0.81	1.04	(0.52–2.07)	0.91
O (42%)	1.05	(0.82–1.36)	0.68	1.04	(0.80–1.34)	0.78
Non-B (89%)	1.00			1.00		
B (11%)	0.85	(0.57–1.27)	0.42	0.89	(0.60–1.32)	0.57
*CCF cohort*						
A (37%)	1.00			1.00		
B (14%)	0.90	(0.79–1.01)	0.08	0.89	(0.79–1.01)	0.06
AB (4%)	0.92	(0.75–1.13)	0.43	0.92	(0.75–1.13)	0.42
O (44%)	0.96	(0.88–1.05)	0.37	0.96	(0.88–1.05)	0.34
Non-B (86%)	1.00			1.00		
B (14%)	0.92	(0.82–1.03)	0.14	0.91	(0.82–1.02)	0.12

^a^All cohorts adjusted for age, sex, ischemic heart disease, previous stroke and whether the patient had a pulmonary infection focus

### Meta-analysis of ABO blood types and mortality

**Fig. 3:**
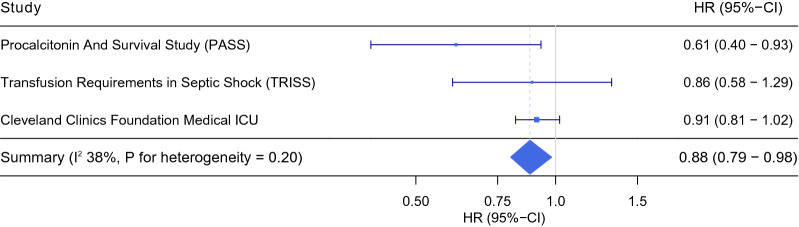
The effect of blood B versus non-blood type B on 30-day mortality in three independent ICU cohorts—individual patient data meta-analysis

We pooled the results from the individual cohorts in an individual patient data meta-analysis (Fig. 3, Table 3). No heterogeneity was detected. Blood type B carried a lower risk of death compared to the non-blood type B pooled, adjusted HR 0.88 (95%-CI 0.80–0.98; *P* = 0.02). When analysed individually, only blood type B had lower mortality, adjusted HR 0.87 (95%-CI 0.77–0.97; *P* = 0.01), while no difference was observed with regard to blood type O and AB. There was no interaction between the site of infection and the effect of ABO blood type on mortality in any of the analyses.

**Table 3:** The effect of ABO blood type on 30-day mortality in critically ill patients—individual patient data meta-analyses

Blood type (percentage of cohort)	Univariable					Multivariable^a^				
	Fixed effect			Heterogeneity		Fixed effect			Heterogeneity	
	HR	(95%-CI)	*p*	*I*^2^ (%)	*p*	HR	(95%-CI)	*p*	*I*^2^ (%)	*p*
A (38%)	1.00					1.00				
B (14%)	0.88	(0.78–0.98)	0.02	13	0.32	0.87	(0.77–0.97)	0.01	16	0.30
O (44%)	0.97	(0.90–1.05)	0.42	0	0.69	0.96	(0.89–1.04)	0.33	0	0.82
AB (4%)	0.98	(0.82–1.18)	0.87	53	0.12	0.99	(0.82–1.18)	0.87	51	0.13
Non-B (86%)	1.00									
B (14%)	0.89	(0.80–0.99)	0.03	48	0.15	0.88	(0.80–0.98)	0.02	38	0.20

^a^All cohorts adjusted for age, sex, ischemic heart disease, previous stroke and whether the patient had a pulmonary infection focus. The analyses are stratified for recruiting centre

### ABO blood type and acute kidney injury

We had data allowing the identification of patients with AKI in the PASS and CCF cohorts. In the PASS cohort, 531/936 (57%) presented with or developed AKI within 4 days of ICU admission. The incidence of AKI across the blood types was A 60%, B 50%, O 55% and AB 59%. In the CCF cohort, 6092/10,549 (58%) developed or presented with AKI. The incidence of AKI across the blood types were A 58%, B 57%, O 58% and AB 62%. There were therefore no clinically meaningful differences in risk of AKI between blood type B and the other blood types, either pooled or individually (Additional file 1: Table S1).

### ABO blood type and endothelial damage

We had access to endothelial damage markers in the PASS cohort in 861/936 (92%) of the included patients. The median (IQR) levels of syndecan-1 according to blood type were: blood type A 181 ng/mL (91–268 ng/mL), blood type B 131 ng/mL (80–224 ng/mL), blood type O 135 ng/mL (78–237 ng/mL), and blood type AB 159 ng/mL (84–223 ng/mL). In adjusted analyses, blood type B and O had lower concentrations than blood type A (Additional file 2: Table S2). When comparing B to non-B blood types no difference was found.

The median levels (IQR) of sTM across the blood types were: blood type A 11.5 ng/mL (8.5–15.4 ng/mL), blood type B 10.5 ng/mL (7.5–13.4 ng/mL), blood type O 11.1 ng/mL (7.5–14.2 ng/mL), and blood type AB 12.4 ng/mL (10.3–16.6 ng/mL). In direct comparison sTM was lower in blood type B and O compared to blood type A (Additional file 2: Table S2). When comparing B to non-B blood types there was no difference.

## Discussion

In this large multicentre, international ICU cohort of septic patients, patients with blood type B were more likely to survive 30 days compared to patients with other blood types although the difference in mortality was limited and showed substantial variation across the included cohorts. Nevertheless, the results were robust to adjustment for known confounding factors. In contrast, we were unable to confirm previous reports of an association between blood type and acute kidney injury. Although markers of endothelial damage were slightly lower with type B than A, no overall difference was evident when comparing type B to all other blood types.

ABO blood types are genetically determined and a well-defined phenotype. In 2015, a higher incidence of AKI was noted in patients suffering sepsis and trauma who had blood type A, and the data suggested a lower mortality in patients with blood type B [9]. This observation suggested a genetic component for development of organ failure in patients with sepsis and associated mortality. Subsequent studies evaluated the effect of ABO blood types on mortality in various populations of critically ill patients. One, for example, included 7,340 unselected critically ill patients found that blood type AB was associated with a reduced risk of death compared to the remaining blood types [11]. It is, however, worth noting that the study was based on unselected ICU patients and did not report the proportion with sepsis, and that only 221 patients (3%) had blood type AB, which may increase the risk of co-incidental findings. That study did not specifically report on the findings for the other blood types. A second recent report found no association between blood type and ICU mortality in 1,743 patients with severe hypoxemic respiratory failure. It is worth noting that blood type B had a numerically lower mortality, and that the fraction of patients with sepsis was not reported [10]. Recently, a correlation with severe pulmonary dysfunction on COVID-19 diseases has also been reported [23, 24].

There are thus diverging results regarding the influence of the ABO blood types on the course of critical illness. Selection bias is an important consideration. We evaluated mortality among patients who had a sepsis diagnosis. But ABO blood type may have influenced underlying predisposition to conditions that promote ICU admission and a sepsis diagnosis. For example, certain blood types may increase the risk of acquiring an organ injury that mandates intensive care such as hypoxemic respiratory failure. Nonetheless, the observed distribution of blood types in all three cohorts were roughly as expected for the regional populations, suggesting that selection bias was not a large factor.

Our results have several important implications for our understanding of critical illness. First, the finding that blood type—a single highly specific genetically determined risk factor—influences the overall mortality of the ICU population with sepsis suggests a common frailty (or resistance) to insults predisposing to critical illness in some patients. Second, the corresponding weak association with biomarkers of endothelial damage, largely for type B versus type A, suggests a potential mechanism. The increased level of endothelial damage in blood type O is however not completely congruent with this, and emphasize the complicate pathology of sepsis There are, however, several possible pathways from ABO blood type to increased susceptibility to endothelial damage: the molecular composition of the blood type may directly damage the endothelium during a critical insult like trauma or sepsis or propagate an otherwise self-limiting damage, and promote, i.e. hypercoagulation syndromes such as disseminated intravascular coagulation as has recently been described [25]. Furthermore, the endothelial cells express blood type antigens that may stabilize or destabilize the endothelium dependent on blood type antigens expressed (and thereby blood type). Finally, the glycosyl-transferring enzyme responsible for the ABO blood type may have other unknown physiological roles. An alternative explanation is that blood type is merely a confounder for other host-defence genes that cluster with blood type. Our results are in line with recent findings from other sepsis populations[25].

This study is observational by nature with consequent limitations on causal inference. However, we have adjusted for several potential confounders of the association between blood type and the outcomes. Although it is difficult to rule out unobserved confounder effects, the random nature of the genetic distribution of blood type genes would require unobserved confounding factors to follow the same distribution. This genetic ‘randomization’ is related to the mechanism used in mendelian randomization studies and would tend to mitigate the effect of unobserved confounders. We did not publish a protocol, but an internal protocol and statistical analysis plan was prepared prior to analysis and data acquisition. We used the most recently available sepsis definition available in each cohort, which could induce clinical heterogeneity.

Blood type may increase the risk of sustaining an organ injury mandating intensive care, and thus adjusting for such variations in the population may remove such effects. We therefore adjusted for medical comorbidities known to influence the clinical course of sepsis and associated with the blood types, age, gender and whether the patients had a pulmonary infection. Furthermore, in post hoc sensitivity analyses, we tested whether there was an interaction between mechanical ventilation and pulmonary infection and the effects of the blood types, but none was observed.

The large sample size and complete follow-up of patients makes random error unlikely. Additionally, congruence between mortality and endothelial function strengthen the biological plausibility of our findings. The CCF MICU and TRISS cohorts were substantially different from the PASS cohort where the most pronounced effect was observed. The TRISS cohort is a much more selected group of patients (septic shock and anaemia) which could make a signal less pronounced as other factors associated with the risk of death may mitigate an effect as discussed previously, and the CCF cohort is a much broader cohort of patients. It is also worth noticing that the PASS and TRISS cohorts are Scandinavian reflecting a mainly Caucasian population, whereas the Cleveland Clinics sepsis cohort is North American and likely more diverse. This could potentially explain some of the variation observed between the cohorts.

The findings of the present and related studies, and the heterogeneity observed between the current cohorts, warrants further investigation. Future studies should focus on further exploring the relation between blood types and endothelial damage, and on pinpointing pathophysiological mechanisms adding additional markers of endothelial damage could potentially aid this. For example, specific genomic influences on sepsis could be evaluated in genome-wide association studies. Future studies should be specifically aimed at elucidating these mechanisms and ensure to include potential confounders, including ethnicity.

## Conclusion

Septic patients with blood type B had less endothelial damage, and a small reduction in mortality. The exposure is, however, unmodifiable.

## Supplementary Information

**Additional file 1.** Risk of AKI within 4 days of ICU admission.

**Additional file 2.** Level of endothelial damage markers across the blood types in the Procalcitonin and Survival Study (PASS) cohort.

## Data Availability

The data used for this publication will not be made available for others due to limitations on the consent from participating patients.

## References

[1] Dean L. ABO Blood Group. Medical Genetics Summaries. 2015;1–3.

[2] Ravn V, Dabelsteen E (2000). Tissue distribution of histo-blood group antigens. APMIS.

[3] Johansson P, Stensballe J, Ostrowski S (2017). Shock induced endotheliopathy (SHINE) in acute critical illness—a unifying pathophysiologic mechanism. Crit Care.

[4] Aird WC (2003). The role of the endothelium in severe sepsis and multiple organ dysfunction syndrome. Blood.

[5] Johansen ME, Johansson PI, Ostrowski SR, Bestle MH, Hein L, Jensen ALG (2015). Profound endothelial damage predicts impending organ failure and death in sepsis. Semin Thromb Hemost.

[6] Hoste EAJ, Bagshaw SM, Bellomo R, Cely CM, Colman R, Cruz DN (2015). Epidemiology of acute kidney injury in critically ill patients: the multinational AKI-EPI study. Intensive Care Med..

[7] Villar J, Blanco J, Anon JM, Santos-Bouza A, Blanch L, Ambros A (2011). The ALIEN study: incidence and outcome of acute respiratory distress syndrome in the era of lung protective ventilation. Intensive Care Med.

[8] Reilly JP, Meyer NJ, Shashaty MGS, Feng R, Lanken PN, Gallop R (2014). ABO blood type a is associated with increased risk of ARDS in whites following both major trauma and severe sepsis. Chest.

[9] Reilly JP, Anderson BJ, Mangalmurti NS, Nguyen TD, Holena DN, Wu Q (2015). The ABO histo-blood group and AKI in critically ill patients with trauma or sepsis. Clin J Am Soc Nephrol.

[10] Rezoagli E, Gatti S, Villa S, Villa G, Muttini S, Rossi F (2018). ABO blood types and major outcomes in patients with acute hypoxaemic respiratory failure: a multicenter retrospective cohort study. PLoS ONE.

[11] Slade R, Alikhan R, Wise MP, Germain L, Stanworth S, Morgan M (2019). Impact of blood group on survival following critical illness: a single-centre retrospective observational study. BMJ Open Respir Res.

[12] Singer M, Deutschman CS, Seymour CW, Shankar-Hari M, Annane D, Bauer M (2016). The Third International Consensus Definitions for Sepsis and Septic Shock (Sepsis-3). JAMA.

[13] Levy MM, Fink MP, Marshall JC, Abraham E, Angus D, Cook D (2003). 2001 SCCM/ESICM/ACCP/ATS/SIS International Sepsis Definitions Conference. Intensive Care Med.

[14] Jensen JU, Hein L, Lundgren B, Bestle MH, Mohr TT, Andersen MH (2011). Procalcitonin-guided interventions against infections to increase early appropriate antibiotics and improve survival in the intensive care unit: a randomized trial. Crit Care Med.

[15] Holst LB, Haase N, Wetterslev J, Wernerman J, Guttormsen AB, Karlsson S (2014). Lower versus higher hemoglobin threshold for transfusion in septic shock. N Engl J Med.

[16] Kidney Disease: Improving Global Outcomes (KDIGO) Acute Kidney Injury Work Group. KDIGO Clinical Practice Guideline for Acute Kidney Injury. Kidney international supplements. 2012;2:1–138.

[17] Itenov TS, Berthelsen RE, Jensen J-U, Gerds TA, Pedersen LM, Strange D (2018). Predicting recovery from acute kidney injury in critically ill patients: development and validation of a prediction model. Crit Care Resusc.

[18] Levey AS, Bosch JP, Lewis JB, Greene T, Rogers N, Roth D. A more accurate method to estimate glomerular filtration rate from serum creatinine: a new prediction equation. Modification of Diet in Renal Disease Study Group. Ann Intern Med. 1999;130:461–70. 10.7326/0003-4819-130-6-199903160-00002 10075613

[19] Jr FEH, Lee KL, Mark DB. Tutorial in biostatistics multivariable prognostic models: issues in developing models, evaluating assumptions and adequacy, and measuring and reducing errors. 1996;15:361–87. 10.1002/(SICI)1097-0258(19960229)15:4<361::AID-SIM168>3.0.CO;2-4 8668867

[20] de Jong VMT, Moons KGM, Riley RD, Tudur Smith C, Marson AG, Eijkemans MJC (2020). Individual participant data meta-analysis of intervention studies with time-to-event outcomes: a review of the methodology and an applied example. Res Synth Methods.

[21] R Core Team. R: A Language and Environment for Statistical Computing. Vienna, Austria: R Foundation for Statistical Computing; 2017. https://www.r-project.org/

[22] Garratty G, Glynn SA, McEntire R (2004). ABO and Rh(D) phenotype frequencies of different racial/ethnic groups in the United States. Transfusion.

[23] Liu Y, Häussinger L, Steinacker JM, Dinse-Lambracht A. Association between the dynamics of the COVID-19 epidemic and ABO blood type distribution. Epidemiol Infect. 2021:1–17. 10.1017/S0950268821000030 PMC7844181 33407977

[24] Li J, Wang X, Chen J, Cai Y, Deng A, Yang M (2020). Association between ABO blood groups and risk of SARS-CoV-2 pneumonia. Br J Haematol.

[25] Reilly JP, Meyer NJ, Shashaty MG, Anderson BJ, Ittner C, Dunn TG (2020). The ABO Histo-Blood Group, endothelial activation, and acute respiratory distress syndrome risk in critical illness. J Clin Invest..

