# Supplementary material for: ABO blood types and sepsis mortality
Source: Ann Intensive Care. 2021 Apr 20;11:61. doi: 10.1186/s13613-021-00844-2 (PMC8056100; doi:10.1186/s13613-021-00844-2)
Supplement: Supplementary file 1 — Additional file 1. Risk of AKI within 4 days of ICU admission. [file 13613_2021_844_MOESM1_ESM.docx]

**Supplementary table 1: Risk of AKI within 4 days of ICU admission - logistic regression**

| **Blood type** | **Univariable** | | | **Multivariable^1^** | | |
| --- | --- | --- | --- | --- | --- | --- |
|  | **Odds ratio** | **(95%-CI)** | **P** | **Odds ratio** | **(95%-CI)** | **P** |
| **PASS cohort** |  |  |  |  |  |  |
| A | 1.00 |  |  | 1.00 |  |  |
| B | 0.68 | (0.44 to 1.05) | 0.08 | 0.67 | (0.43 to 1.04) | 0.08 |
| O | 0.84 | (0.63 to 1.11) | 0.22 | 0.81 | (0.61 to 1.08) | 0.15 |
| AB | 0.98 | (0.50 to 1.90) | 0.94 | 0.92 | (0.47 to 1.81) | 0.82 |
|  |  |  |  |  |  |  |
| Non-B | 1.00 |  |  | 1.00 |  |  |
| B | 0.74 | (0.49 to 1.11) | 0.15 | 0.74 | (0.49 to 1.13) | 0.16 |
|  |  |  |  |  |  |  |
| **CCF cohort** |  |  |  |  |  |  |
| A | 1.00 |  |  | 1.00 |  |  |
| B | 1.06 | (0.94 to 1.19) | 0.38 | 1.07 | (0.95 to 1.20) | 0.31 |
| O | 1.02 | (0.94 to 1.12) | 0.60 | 1.01 | (0.99 to 1.03) | 0.49 |
| AB | 1.20 | (0.98 to 1.47) | 0.08 | 1.05 | (0.99 to 1.10) | 0.06 |
|  |  |  |  |  |  |  |
| Non-B | 1.00 |  |  | 1.00 |  |  |
| B | 1.03 | (0.93 to 1.15) | 0.56 | 1.04 | (0.93 to 1.16) | 0.51 |

^1^Adjusted for age, sex, ischemic heart disease, previous stroke and whether the patient had a pulmonary infection focus.
