# Supplementary material for: ABO blood types and sepsis mortality
Source: Ann Intensive Care. 2021 Apr 20;11:61. doi: 10.1186/s13613-021-00844-2 (PMC8056100; doi:10.1186/s13613-021-00844-2)
Supplement: Supplementary file 2 — Additional file 2. Level of endothelial damage markers across the blood types in the Procalcitonin and Survival Study (PASS) cohort. [file 13613_2021_844_MOESM2_ESM.docx]

**Supplementary table 2: Level of endothelial damage markers across the blood types in the Procalcitonin and Survival Study (PASS) cohort - linear regression**

| **Blood type** | **Univariable** | | | |  | **Multivariable^1^** | | | | |
| --- | --- | --- | --- | --- | --- | --- | --- | --- | --- | --- |
|  | **Mean difference**  **(ng/mL)** | **(95%-CI)** | **P** |  |  | | **Mean difference**  **(ng/mL)** | **(95%-CI)** | | **P** |
| **Syndecan** |  |  |  | |  |  | | |  |  |
| A (reference, median) | 131 |  |  | |  | 131 | | |  |  |
| B | -27.8 | (-51.7 to -3.9) | 0.02 | |  | -27.0 | | | (-50.9 to -2.9) | 0.03 |
| O | -27.7 | (-12.2 to -43.1) | < 0.001 | |  | -27.5 | | | (-43.0 to -12.0) | < 0.001 |
| AB | -22.8 | (-58.2 to 12.5) | 0.20 | |  | -21.2 | | | (-56.6 to 14.2) | 0.24 |
|  |  |  |  | |  |  | | |  |  |
| Non-B (reference, median) | 162 |  |  | |  | 162 | | |  |  |
| B | -14.2 | (-37.1 to 8.6) | 0.22 | |  | -13.4 | | | (-36,3.1 to 9.5) | 0.25 |
|  |  |  |  | |  |  | | |  |  |
| **Thrombomodulin** |  |  |  | |  |  | | |  |  |
| A (reference, median) | 11.5 |  |  | |  | 11.5 | | |  |  |
| B | -1.34 | (-2.55 to -0.13) | 0.03 | |  | -1.29 | | | (-2.49 to -0.08) | 0.04 |
| O | -0.93 | (-1.71 to -0.15) | 0.02 | |  | -0.94 | | | (-1.72 to -0.16) | 0.02 |
| AB | 0.65 | (-1.14 to 2.44) | 0.48 | |  | 0.55 | | | (-1.23 to 2.33) | 0.54 |
|  |  |  |  | |  |  | | |  |  |
| Non-B(reference, median) | 11.0 |  |  | |  | 11.0 | | |  |  |
| B | -0.96 | (-2.11 to -0.20) | 0.10 | |  | -0.88 | | | (-2.03 to 0.27) | 0.13 |

^1^Adjusted for age, sex, ischemic heart disease, previous stroke and whether the patient had a pulmonary infection focus.
